# Abstract of the Proceedings of the Racine Medical Association

**Published:** 1863-02

**Authors:** John G. Meachem

**Affiliations:** Secretary


ART. IV.—*Abstract of the Proceedings of the Racine Medical Association.*

The January meeting was held at the office of the president, Dr. John L. Paige. Members present, Drs. Paige, Hoy, Wadsworth, Thompson, Peak, Nettleton and Meachem.

After the transaction of some preleminary business, Dr. Wadsworth made some remarks on variola. He said as it was reported to be quite prevalent, it would be well for the Association to give the subject some consideration. It was a terribly disgusting disease, and time spent in deriving means for arresting its spread, or ameliorating the sufferings of those unfortunately afflicted with it, was, in his judgment, time *well spent.* If members were treating cases, he hoped they would state to the Association, the number and their character.

Dr. Thompson said he had no case under treatment.

Dr. Hoy was treating one case which was severely confluent. The patient had been almost entirely comatose from the beginning of the suppurative stage. He had been obliged to be liberal in his use of stimulants tonics and nutriment. He was now recovering. He had treated one other of the distinct variety, and three or four cases of varioloid.

Dr. Meachem had treated one case, which was now convalescent, and had seen three of those mentioned by Dr. Hoy.

Dr. Peak asked how early in an attack of variola could the contagion be communicated.

Dr. Hoy thought it could be as early as the eruption began to make its appearance.

Dr. Wadsworth had known two unmistakeable cases where it was communicated during the primary fever, and before any eruption appeared.—He wished some member, if he knew, would state the best mode of preserving vaccine virus in a reliable condition.

Dr. Meachem stated, that the scale could be preserved for years, and maintain its specific powers unchanged, if it was thoroughly excluded from the atmosphere, by being thickly encased in wax, and kept in a cellar, where the temperature would vary but little from 40 degrees. A very high or a very low temperature would destroy its specific qualities.

A case of considerable interest had occurred to him recently, where the vaccine virus had remained in operation in the system for a period of sixteen days. The patient had been exposed to measles, but was not taken ill with the disease, until nine days after the vaccination. The vaccine virus did not begin to work until the rubeolus rash had entirely disappeared.—Scarlatina is known to produce the same delaying effect upon the action of the vaccine matter.

Dr. Page related the particulars of a very remarkable case of dilatation and distension of the rectum, from fæcal accumulations. It filled almost the entire pelvic cavity.

Dr. Hoy was treating a lady for purpura hæmorrhagica, who was near the termination of her period of gestation. There was almost constant oosing of blood from the mucuous surfaces of the mouth and nostrils, and the body was covered with large petechiae. He would ask the Association the best course of treatment to be pursued in the case.

Dr. Page spoke at some length in answer; said he had seen many similar cases during his long practice, and had found the oil of terebinth, given in emulsion, to be by far the best remedy he had ever used. Concentrated nourishment, tonics and stimulants, constituted our main reliance in these cases. If she remained in this condition at parturition, he had no doubt she would sink immediately after. He hoped Dr. Hoy would report further upon this case at the next meeting.

Dr. Wadsworth concurred with Dr. Page by stating that he had more confidence in the turpentine than any other one remedy.

The Association then adjourned to meet on the evening of the second Tuesday of February, at the office of Dr. Meachem.

John G. Meachem, M. D.,
Secretary.

